# Misclassification of females and males in cardiovascular magnetic resonance parametric mapping: the importance of sex-specific normal ranges for diagnosis of health vs. disease

**DOI:** 10.1093/ehjci/jead247

**Published:** 2023-10-04

**Authors:** Katharine E Thomas, Elena Lukaschuk, Mayooran Shanmuganathan, Jamie A Kitt, Iulia A Popescu, Stefan Neubauer, Stefan K Piechnik, Vanessa M Ferreira

**Affiliations:** Division of Cardiovascular Medicine, Radcliffe Department of Medicine, Oxford Centre for Clinical Magnetic Resonance Research (OCMR), University of Oxford, Level 0, John Radcliffe Hospital, Headley Way, Oxford OX3 9DU, UK; Division of Cardiovascular Medicine, Radcliffe Department of Medicine, Oxford Centre for Clinical Magnetic Resonance Research (OCMR), University of Oxford, Level 0, John Radcliffe Hospital, Headley Way, Oxford OX3 9DU, UK; Division of Cardiovascular Medicine, Radcliffe Department of Medicine, Oxford Centre for Clinical Magnetic Resonance Research (OCMR), University of Oxford, Level 0, John Radcliffe Hospital, Headley Way, Oxford OX3 9DU, UK; Division of Cardiovascular Medicine, Radcliffe Department of Medicine, Oxford Centre for Clinical Magnetic Resonance Research (OCMR), University of Oxford, Level 0, John Radcliffe Hospital, Headley Way, Oxford OX3 9DU, UK; Division of Cardiovascular Medicine, Radcliffe Department of Medicine, Oxford Centre for Clinical Magnetic Resonance Research (OCMR), University of Oxford, Level 0, John Radcliffe Hospital, Headley Way, Oxford OX3 9DU, UK; Division of Cardiovascular Medicine, Radcliffe Department of Medicine, Oxford Centre for Clinical Magnetic Resonance Research (OCMR), University of Oxford, Level 0, John Radcliffe Hospital, Headley Way, Oxford OX3 9DU, UK; Division of Cardiovascular Medicine, Radcliffe Department of Medicine, Oxford Centre for Clinical Magnetic Resonance Research (OCMR), University of Oxford, Level 0, John Radcliffe Hospital, Headley Way, Oxford OX3 9DU, UK; Division of Cardiovascular Medicine, Radcliffe Department of Medicine, Oxford Centre for Clinical Magnetic Resonance Research (OCMR), University of Oxford, Level 0, John Radcliffe Hospital, Headley Way, Oxford OX3 9DU, UK

**Keywords:** CMR, parametric mapping, reference range, female, male, sex specific

## Abstract

**Aims:**

Cardiovascular magnetic resonance parametric mapping enables non-invasive quantitative myocardial tissue characterization. Human myocardium has normal ranges of T1 and T2 values, deviation from which may indicate disease or change in physiology. Normal myocardial T1 and T2 values are affected by biological sex. Consequently, normal ranges created with insufficient numbers of each sex may result in sampling biases, misclassification of healthy values vs. disease, and even misdiagnoses. In this study, we investigated the impact of using male normal ranges for classifying female cases as normal or abnormal (and vice versa).

**Methods and results:**

One hundred and forty-two healthy volunteers (male and female) were scanned on two Siemens 3T MR systems, providing averaged global myocardial T1 and T2 values on a per-subject basis. The Monte Carlo method was used to generate simulated normal ranges from these values to estimate the statistical accuracy of classifying healthy female or male cases correctly as ‘normal’ when using sex-specific vs. mixed-sex normal ranges. The normal male and female T1- and T2-mapping values were significantly different by sex, after adjusting for age and heart rate.

**Conclusion:**

Using 15 healthy volunteers who are not sex specific to establish a normal range resulted in a typical misclassification of up to 36% of healthy females and 37% of healthy males as having abnormal T1 values and up to 16% of healthy females and 12% of healthy males as having abnormal T2 values. This paper highlights the potential adverse impact on diagnostic accuracy that can occur when local normal ranges contain insufficient numbers of both sexes. Sex-specific reference ranges should thus be routinely adopted in clinical practice.

## Graphical Abstract

**Graphical Abstract:**
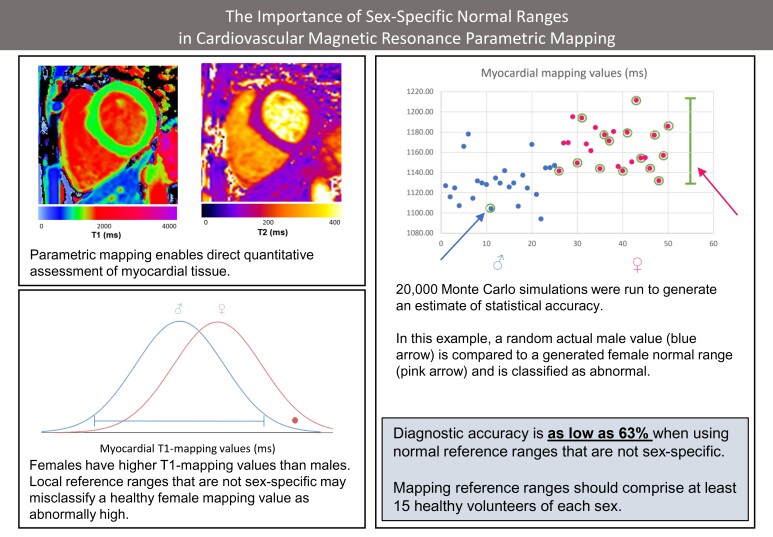

## Introduction

Cardiovascular magnetic resonance (CMR) parametric mapping enables a direct quantitative assessment of myocardial tissue characteristics; T1 and T2 maps measure T1 and T2 relaxation times of tissues on a pixel-by-pixel basis.^1^ Human myocardium has normal ranges of T1 and T2 values, deviation from which may indicate disease or altered physiology. Normal myocardial T1 and T2 values are dependent on numerous technical factors such as magnetic field strength, manufacturer, the mapping method used, and other software parameters.^1–3^ They are also influenced by biophysiological factors such as heart rate, age, and sex.^4–13^

The CMR community recognizes that using a normal range that is not tailored for the specific technique can lead to an inaccurate diagnosis of health vs. disease.^14^ The Society for Cardiovascular Magnetic Resonance (SCMR) mapping consensus statement recommends that each centre establishes local reference ranges using a minimum of 15 healthy volunteers.^1^ It is known that women exhibit higher normal myocardial T1 and T2 values compared with men,^4,10–12,15^ and this may, in fact, be the most significant independent biological factor affecting normal myocardial T1 and T2 values.^6^ Thus, the local normal ranges based on insufficient numbers of each sex may be subject to sampling biases, leading to reduced diagnostic accuracy and potential misdiagnosis.

Given the above, we investigated the impact of using male normal ranges for classifying female cases as normal or abnormal (and vice versa). We also investigated the impact of scanner-specific, rather than centre-specific, reference ranges, even when using magnetic resonance (MR) scanners made by the same manufacturer, with the same magnetic field strength and mapping method parameters.

## Methods

Healthy volunteers (male and female, *n* = 142) were scanned on a Siemens 3T Trio MR system (*n* = 92) and a Siemens 3T Prisma MR system (*n* = 50) (*Table 1*). The volunteers were predominantly White, had no history of cardiovascular conditions, had no cardiac risk factors, and took no regular medication.

**Table 1:** Healthy volunteers’ demographics and their myocardial mapping values categorized by MR scanner and by sex

	Siemens 3T Trio T1 mapping (ShMOLLI WIP 561)		Siemens 3T Prisma T1 mapping (ShMOLLI WIP 1048B)		Siemens 3T Prisma T2 mapping (Myomaps T2 FLASH)	
	Male	Female	Male	Female	Male	Female
Demographics						
Number of participants, *n*	57*	35*	25	25	25	25
Age, years	41 ± 14*	47 ± 14*	34 ± 5	34 ± 7	34 ± 5	34 ± 7
Height (cm)	178.1 ± 8.2*	166.1 ± 6.2*	179.0 ± 5.7*	167.9 ± 6.0*	179.0 ± 5.7*	167.9 ± 6.0*
Weight (kg)	78.6 ± 12.1*	66.6 ± 11.8*	80.3 ± 9.0*	66.5 ± 9.8*	80.3 ± 9.0*	66.5 ± 9.8*
Body mass index (kg/m^2^)	24.7 ± 3.1	24.1 ± 3.7	25.1 ± 2.5	23.6 ± 3.6	25.1 ± 2.5	23.6 ± 3.6
Heart rate (bpm)	57.6 ± 10.9	60.0 ± 12.0	69.3 ± 12.6	65.4 ± 10.2	69.3 ± 12.6	65.4 ± 10.2
Average myocardial mapping values (ms)	1173 ± 28*	1201 ± 25*	1131 ± 20*	1166 ± 20*	39.66 ± 1.98*	41.22 ± 1.96*

All demographic data are presented as mean ± SD. Significant differences between males and females on a per-scanner basis are marked with * (the significance level is set at *P* < 0.05).

**Figure 1:**
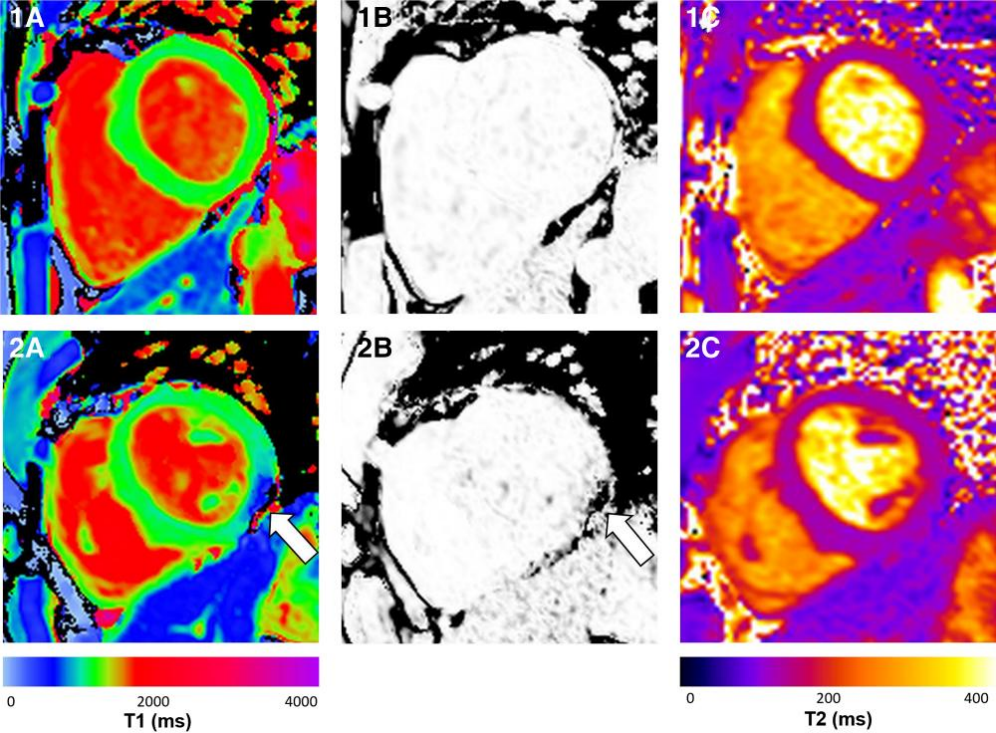
Representative native short-axis myocardial maps from the hearts of two healthy participants. (*A*) ShMOLLI T1 map. (*B*) Parametric goodness-of-fit (*R*^2^) map. (*C*) Myomaps T2-prepared FLASH map. Participant 1 has good-quality parametric maps (*1A* and *1C*), with a good-quality parametric goodness-of-fit (*R*^2^) map, signifying a near-excellent fit between T1-mapping measurements and the ideal signal recovery model predictions (*1B*). Participant 2 has an artefact affecting the inferior and inferolateral segments (white arrow) in their T1 map (*2A*), with a corresponding poor-quality parametric goodness-of-fit (*R*^2^) map in the affected segments (white arrow) (*2B*).

Whole-heart, short-axis, native myocardial T1 and T2 maps were acquired using Siemens Work-In-Progress (WIP) prototypes of the Shortened Modified Look-Locker Inversion (ShMOLLI) recovery sequence^16^ and Myomaps T2-prepared fast low-angle shot (FLASH) in the Prisma cohort (*Figure 1*). In the Trio cohort, at least one short-axis, mid-ventricular ShMOLLI T1 map was acquired per subject. Endocardial and epicardial borders were manually contoured on the left ventricular (LV) myocardium using cvi42 (Circle Cardiovascular Imaging, Calgary, Canada). Each slice was segmented into six segments. All maps were checked for artefacts, including parametric goodness-of-fit (*R*^2^) maps that were available for ShMOLLI T1 maps,^1,17,18^ and any affected segments were excluded. Following quality control, the remaining segments were used to generate average global myocardial T1 and T2 values on a per-subject basis.

The normality of data was confirmed using the Kolmogorov–Smirnov test. Comparisons of means between independent samples were made using unpaired *t*-tests. Multiple comparisons of age, heart rate, and body mass index (BMI) across both scanners were made using analysis of variance. Bartlett’s test was used to test for any significance of variation between groups, and *χ*^2^ tests were used to assess whether the distribution of sex within each cohort matched that of the general population and in the assessment of the frequency of artefacts between sexes. Multiple linear regression was performed to investigate the relationship between T1- and T2-mapping values and potentially confounding factors such as age, sex, heart rate, and BMI. All tests were two-tailed, with a significance level set at *P* < 0.05.

The Monte Carlo method was used to run 20 000 simulations whereby the T1 and T2 reference ranges were generated using any random 15 participant combinations from each cohort (male-only, female-only, or mixed sex). The sex-specific actual T1 and T2 values were compared with the generated normal ranges [simulated mean ± 2 standard deviations (SDs)] to estimate the diagnostic accuracy of local reference ranges in defining whether healthy males and females were correctly identified as normal.

To optimize diagnostic accuracy, the generated mixed-sex ranges were drawn from balanced groups of men and women. Given that there were significantly more men than women for the Trio scanner data (male = 57, female = 35), for each Monte Carlo simulation, 35 male T1 values were selected randomly from the 57 possible male T1 values and then added to the 35 female T1 values. Any random 15 T1 values were then selected from these 70 values (male = 35, female = 35) in order to create the T1 and T2 ranges. The Prisma scanner dataset was balanced (male = 25, female = 25).

## Results

### Per-scanner results of myocardial T1 and T2 values by sex

**Figure 2:**
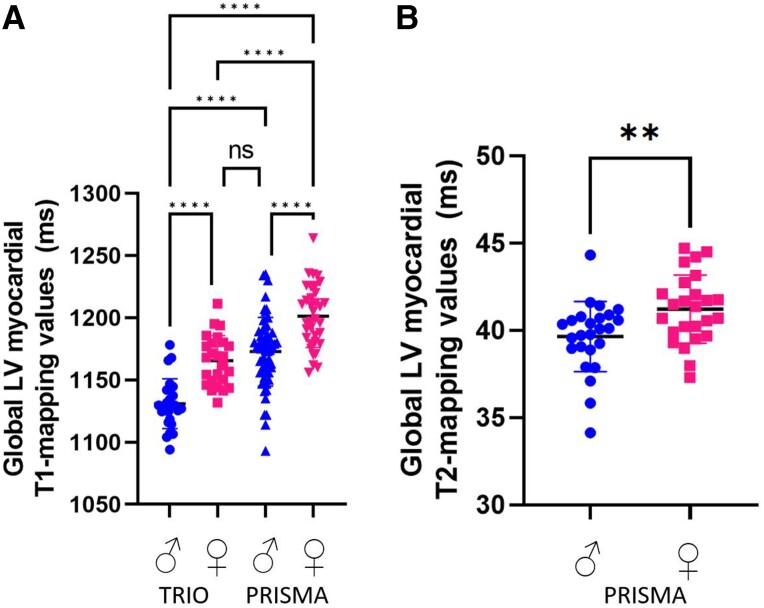
Native LV myocardial (*A*) T1-mapping values and (*B*) T2-mapping values for male and female healthy volunteers on the Siemens 3T Prisma and the Siemens 3T Trio MR scanners. Females show significantly higher T1 and T2 values than males. The significance level is set at *P* < 0.05.

Female myocardial mapping values were higher than male values for both T1 mapping (Trio *P* < 0.0001; Prisma *P* < 0.0001, unadjusted) and T2 mapping (*P* = 0.007, Prisma, unadjusted). After adjusting for age, BMI, and heart rate using multiple linear regression, women still had significantly higher T1 values (Trio *P* < 0.0005; Prisma *P* < 0.0005) and T2 values (Prisma *P* = 0.029) than men (*Figure 2*). These findings are consistent with those of previous literature.^4,15^ Multiple regression analysis revealed that T1 and T2 values were significantly correlated with sex and heart rate but not with BMI or age (*Table 2*).

**Table 2:** A multiple regression analysis examining the effect of sex, heart rate, age, and BMI on T1- and T2-mapping values categorized by MR scanner

	Siemens 3T Trio T1 mapping (ShMOLLI WIP 561)		Siemens 3T Prisma T1 mapping (ShMOLLI WIP 1048B)		Siemens 3T Prisma T2 mapping (Myomaps T2 FLASH)	
	*β*	*P*	*β*	*P*	*β*	*P*
Sex [ms/(male/female)]	−27.253	<0.0005*	−38.834	<0.0005*	−1.224	0.029*
Heart rate (ms/bpm)	0.672	0.007*	0.943	<0.0005*	−0.074	0.003*
Age (ms/years)	−0.075	0.711	0.834	0.056	0.036	0.440
Body mass index (ms/kg/m^2^)	−0.089	0.915	0.004	0.996	−0.054	0.779

*P < 0.05.

There were balanced numbers of men and women within the Prisma cohort (*P* = 1.00), but significantly more men than women in the Trio cohort (*P* = 0.022). There were significant differences between men and women in height (*P* < 0.0001; both scanners) and weight (*P* < 0.0001; both scanners). However, BMI was not significantly different (Trio *P* = 0.37; Prisma *P* = 0.29). There was no significant difference in the heart rate between men and women on either scanner (Trio *P* = 0.31; Prisma *P* = 0.21). Women were significantly older than men in the Trio cohort (*P* = 0.048). However, age did not differ significantly between men and women in the Prisma cohort (*P* = 0.74).

Using only males or only females to create local normal ranges leads to significantly reduced diagnostic accuracy of normal healthy cases for both T1 and T2 mapping (*Table 3*). When using male-only reference ranges for healthy women, 14–36% of female T1 values and 16% of female T2 values were incorrectly classified as abnormal. Similarly, when using female-only reference ranges for healthy men, 19–36% of male T1 values and 12% of male T2 values were incorrectly classified as abnormal (*Table 3*).

**Table 3:** Diagnostic accuracy estimated by the rate of correct identification of actual normal male or female mapping values when compared with generated normal ranges (20 000 possible permutations via Monte Carlo simulation)

	Siemens 3T Trio T1 mapping (ShMOLLI WIP 561)		Siemens 3T Prisma T1 mapping (ShMOLLI WIP 1048B)		Siemens 3T Prisma T2 mapping (Myomaps T2 FLASH)	
	Male	Female	Male	Female	Male	Female
Diagnostic accuracy—same scanner						
Using any random 15 participants (mixed sex; same scanner)	93% (88–96)	97% (97–97)	96% (96–100)	96% (88–96)	92% (88–96)	96% (88–100)
Using any random 15 participants (opposite sex; same scanner)	81% (74–86)	86% (74–94)	64% (52–72)	64% (48–64)	88% (88–92)	84% (84–88)
Diagnostic accuracy—cross-scanner						
Using any random 15 participants (mixed sex; cross-scanner)	86% (82–91)	51% (40–54)	56% (28–76)	100% (96–100)		
Using any random 15 participants (opposite sex; cross-scanner)	84% (81–86)	12% (8–16)	12% (12–16)	100% (100–100)		

Mixed-sex ranges drawn from balanced numbers of males and females. All data are presented as median (inter-quartile range).

### Cross-scanner comparisons of normal myocardial T1 and T2 values by sex

Across all four cohorts (Trio men and women and Prisma men and women), there was a significant difference in age (*P* < 0.0001) and heart rate (*P* < 0.0001), but not in BMI (*P* = 0.353). There was a significant difference in normal myocardial T1 values between the two Siemens 3T scanners, even when adjusting for age, heart rate, and BMI (*P* < 0.0001).

Applying the normal T1-mapping reference range from one MR scanner to cases obtained on a different MR scanner (same manufacturer, field strength, software, and T1-mapping methodology) grossly affected cross-scanner accuracy (12–100%).

### Assessment of partial volume effect in T1 maps

A comparison of T1-mapping values in 20 participants (10 men and 10 women, Prisma cohort) between the mid-ventricular septum and the globally averaged T1-mapping value demonstrated a decrease of 0.78% for female T1 values and 0.32% for male T1 values in the mid-ventricular septum. The mid-ventricular septum was chosen as this is usually the thickest section of the myocardium, which is unlikely to be affected by the left ventricular outflow tract or thinner apical walls, and therefore, is less susceptible to the partial volume effect. Also, there is a much lower likelihood of the presence of artefacts within the septal segments.

### Frequency and location of artefacts

**Figure 3:**
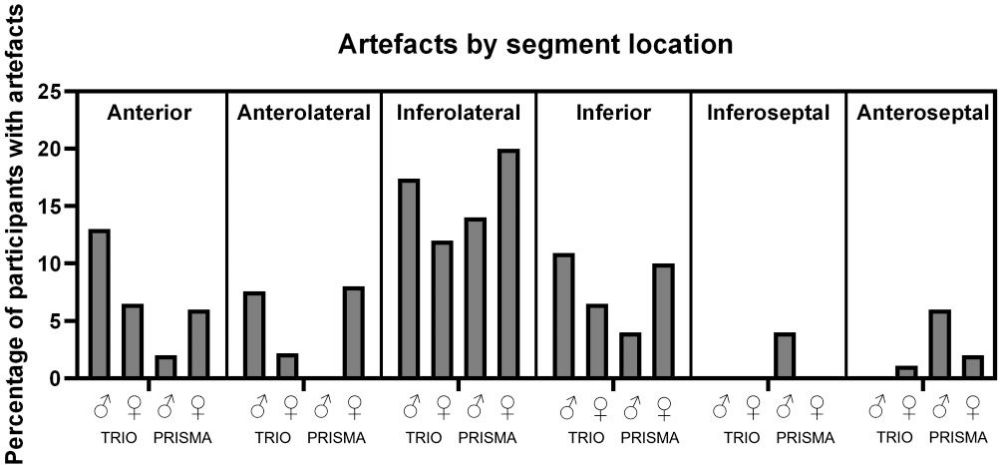
Percentage of participants with artefacts seen on T1 mapping. Slices were divided into six segments, and each segment was checked for artefacts. Artefact data are presented by segment location and subdivided by scanner (Trio or Prisma) and sex (male ♂ or female ♀).

There was exclusion of segments due to the presence of artefacts in 8.1% of segments (187/2316 segments; Prisma females 10.5%, males 6.1%; Trio females 12.4%, males 13.2%), which is consistent with that of previous literature.^10,17,19^ Artefacts were significantly more likely to be present in females than in males in the Prisma cohort but were not significantly more likely in the Trio cohort (Prisma whole-heart *P* = 0.048, mid-ventricular *P* = 0.042; Trio females *P* = 0.347). The most common location for artefacts was within the inferolateral wall for both males and females, although artefacts can be seen in other LV myocardial walls (*Figure 3*). In terms of the actual location of artefacts, there was no significant difference between males and females (anterior *P* = 0.890; anterolateral *P* = 0.997; inferolateral *P* = 0.376; inferior *P* = 0.719; inferoseptal *P* = 0.619; and anteroseptal *P* = 0.917).

## Discussion

There is growing awareness that sex-specific reference ranges are required for a number of CMR techniques, including parametric mapping and quantitative myocardial perfusion mapping.^6,20^ Current recommendations for CMR parametric T1 and T2 mapping are to establish local normal ranges with a minimum of 15 healthy volunteers.^1^ These volunteers do not have to be sex specific. This study demonstrates that following current recommendations would lead to a misclassification of up to 36% of healthy females and 37% of healthy males as having abnormal T1 values and up to 16% of healthy females and 12% of healthy males as having abnormal T2 values. Our data demonstrate the importance of adopting sex-specific normal reference ranges in clinical practice for myocardial T1 and T2 mapping to improve diagnostic accuracy and provide an estimate of the negative impact on diagnostic accuracy that can occur when local reference ranges do not contain sufficient numbers of both men and women.

### Differences between males and females

Our results confirm that women have higher myocardial T1- and T2-mapping values than men, even after accounting for confounding factors such as heart rate and age.

On a per-scanner basis, the diagnostic accuracy was lower for the Siemens 3T Prisma scanner (64%) compared with the Siemens 3T Trio scanner (81–86%) when failing to use sex-specific reference ranges. This difference may be explained by the Prisma having a more balanced healthy volunteer cohort (no significant difference in age, heart rate, or BMI between men and women) than the Trio cohort. Notably, the findings described here are based on typical (median) observed accuracy. When lower quartiles are considered (i.e. a one-in-four chance), even centres following current SCMR recommendations may encounter diagnostic accuracies as low as 52%.

#### Heart rate effect on myocardial T1 and T2 values

Women tend to have higher resting heart rates than men,^21^ although our cohorts did not show a significant difference in heart rate between men and women. Physiologically, higher resting heart rates are correlated with higher myocardial T1 value and lower myocardial T2 value.^4,22–25^ However, multiple regression analysis reveals that female T1- and T2-mapping values are significantly higher than male T1- and T2-mapping values even after adjusting for heart rate. The effect of heart rate on myocardial T1 values is small (around 6 ms per 10 bpm)^4^, and is, therefore, not sufficient to explain the ∼30 ms T1-mapping difference seen between the two sexes.

#### Partial volume effect on myocardial T1 and T2 values

There may be a greater partial volume effect in women due to thinner myocardial walls,^4,26^ which may lead to an overestimation of true female myocardial T1 and T2 values. However, data from our study and others show that contour erosion (to account for the partial volume effect) leads to a decrease of only 0.3–1% in mapping values.^4^ Any overestimation of female mapping values due to thinner myocardial walls is likely to be minimal.

#### Age

Our analysis showed that age did not correlate with T1- or T2-mapping values, which contrasts with that of previous studies.^4,5,7,15,22,27^ It is possible that the age range was too narrow, and the cohort numbers too small, to demonstrate a significant effect. However, linear regression analysis suggests that age was not a confounding factor for this dataset.

#### Frequency and location of artefacts

Significantly, there was a greater exclusion of segments due to the presence of artefacts in females than in males in our Prisma cohort but not in our Trio cohort. As the female myocardium is thinner than the male myocardium,^4,26^ contour erosion to avoid artefact is often less successful, leading to higher rates of segment exclusion in the Prisma cohort. The difference may be non-significant between sexes in the Trio because of it being an older machine with less effective shimming, which leads to a higher rate of artefacts overall.

Artefacts were seen most frequently in the inferolateral wall, which has been reported by previous studies.^10,24^ However, there was no significant difference in the location of artefacts by sex. This would suggest that globally averaged T1 values of males and females contain similar areas of the myocardium, rather than a significant exclusion of one particular area of myocardium by sex.

### Differences between MR scanners

Small but significant inter-version drifts in normal T1 values within the same scanner manufacturer have been described previously,^28^ even with identical ShMOLLI acquisition parameters. These results illustrate the importance of local scanners and sex-specific reference ranges, even when using MR scanners made by the same manufacturer and of the same field strength.

#### Differences in iterative versions of a mapping sequence

Two different prototypes of the ShMOLLI sequence are presented in this paper: ShMOLLI WIP 561 and ShMOLLI WIP 1048B. Although both use the same fundamental ShMOLLI acquisition parameters and protocol [9 heartbeats 5(1)1(1)1 acquisition], ShMOLLI WIP 561 is an older ShMOLLI prototype, which includes a 10 ms duration of the adiabatic inversion pulse within its inversion time calculation. The manufacturer has updated this in newer versions (including WIP 1048B), but these versions require up to 25 ms correction to obtain equivalent values.^28^

#### Different healthy volunteer populations

The healthy volunteers in this study were only a subset of the wider population, and a cross-scanner comparison revealed that they had significantly different ages and heart rates. These sources of variation will contribute to the difference seen in mean mapping values on a per-group basis and therefore contribute to the varying diagnostic accuracies seen in cross-scanner comparisons.

A precise allocation of the underlying cause of inter-scanner difference requires a significant amount of reverse engineering, which is beyond the scope of this paper.

### Standardization and quality control

Many factors affect myocardial T1 values, both biophysiological and technical.^1,4^ The Consensus statement therefore recommends that each centre establish local reference ranges to ensure diagnostic accuracy.^1^

We have demonstrated that larger cohorts of healthy volunteers of both sexes are required to mitigate sampling bias. However, many centres often find it difficult to establish larger cohorts because of limitations on time and resources,^29^ and therefore, there is a need for finding more practical solutions such as phantom quality control and standardization.^30^

Phantoms are standard reference objects that can be used to provide calibration or validation of technical methods^31^ and can be used to ensure local accurate calibration of mapping methods. Quality assurance programmes use data from phantoms to ensure adequate technical tolerances for myocardial T1-mapping method deployment,^30^ which promotes T1-mapping consistency and method stability across a number of different centres.

Sampling biases could, therefore, be minimized through a combination of local reference ranges of healthy volunteers and quality assurance and standardization with phantoms.

## Conclusion

We propose that local T1- and T2-mapping reference ranges should comprise at least 15 healthy volunteers of each sex and be generated on a per-scanner basis, to improve diagnostic accuracy for both females and males. Larger numbers of healthy volunteers help to mitigate sampling bias encountered when using a smaller number of healthy volunteers, but larger cohorts of healthy volunteers can be impractical in smaller centres with more limited resources. Thus, more practical solutions, such as phantom quality control and standardization, are needed.^1^

## Data Availability

The data underlying this article will be shared on reasonable request to the corresponding author.
